# Supplementary figures and images for: A Rad51-independent pathway promotes single-strand template repair in gene editing
Source: PLoS Genet. 2020 Oct 15;16(10):e1008689. doi: 10.1371/journal.pgen.1008689 (PMC7591047; doi:10.1371/journal.pgen.1008689)

S1 Fig

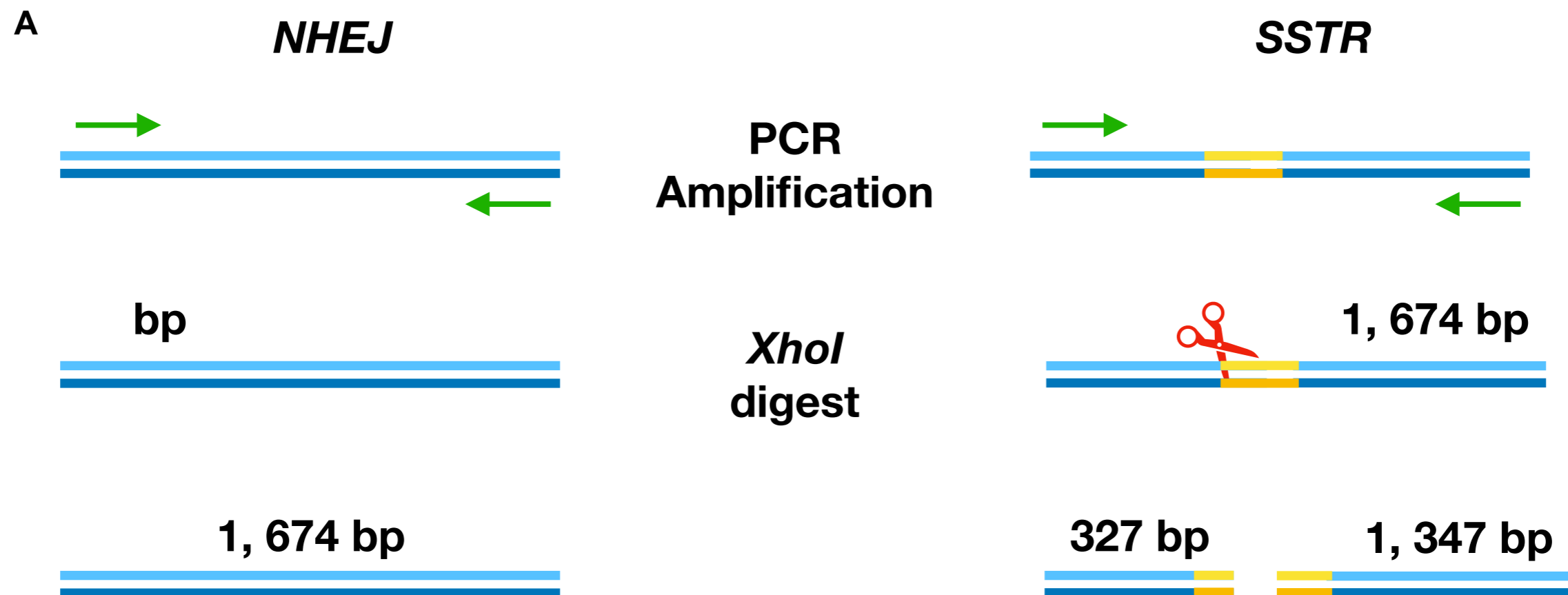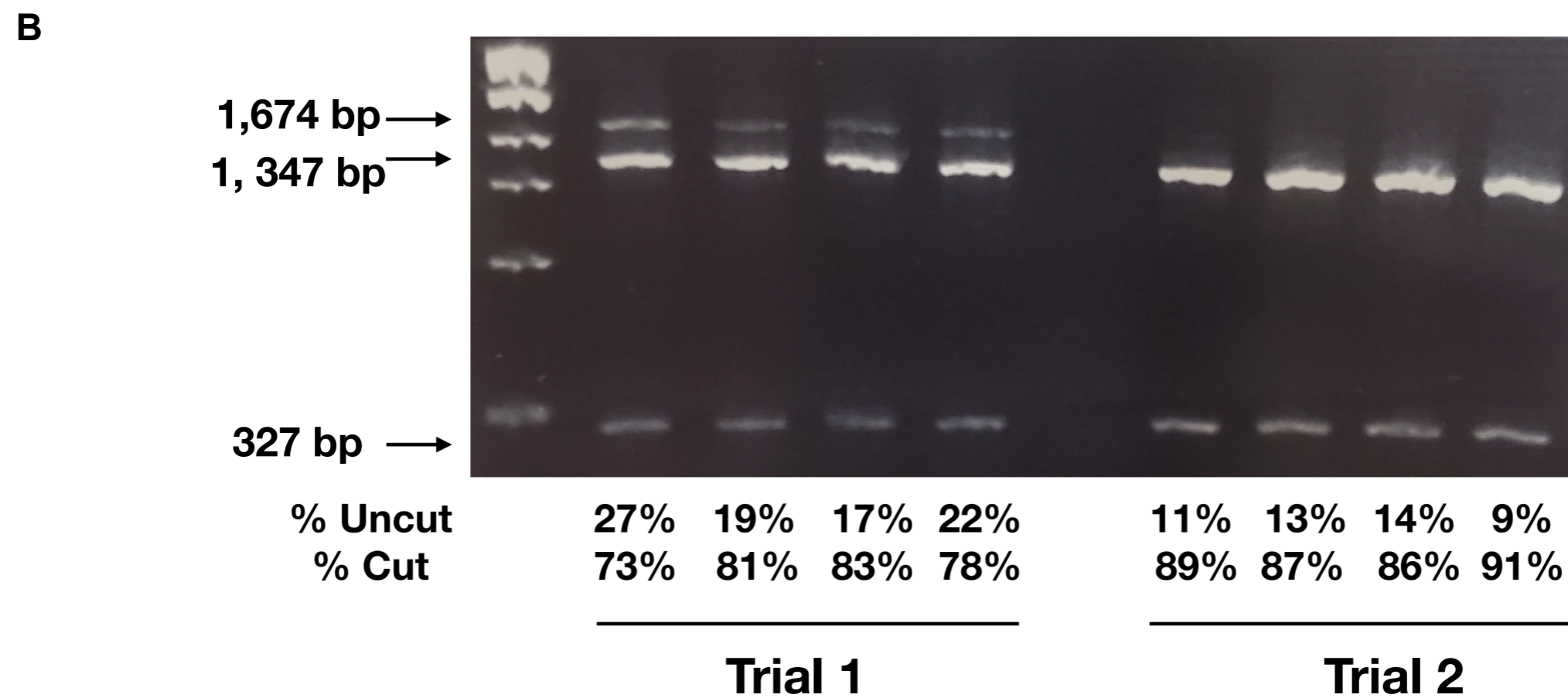

Supplement: S1 Fig — The percentage of SSTR after the experiment described in Fig 1 was determined by PCR across the MAT locus using primers DG_253 and DG_254 (S2 Table), followed by XhoI restriction digest. NHEJ events will result in a non-digested product of 1,674 bp, while SSTR results in two bands at 1,347 bp and 327 bp. The intensity of the bands was quantified as shown by Gel Doc Imager. Trial 1 and 2 were performed on different days with different sets of media, but with identical protocols. (PDF) [file pgen.1008689.s001.pdf]

S2 Fig

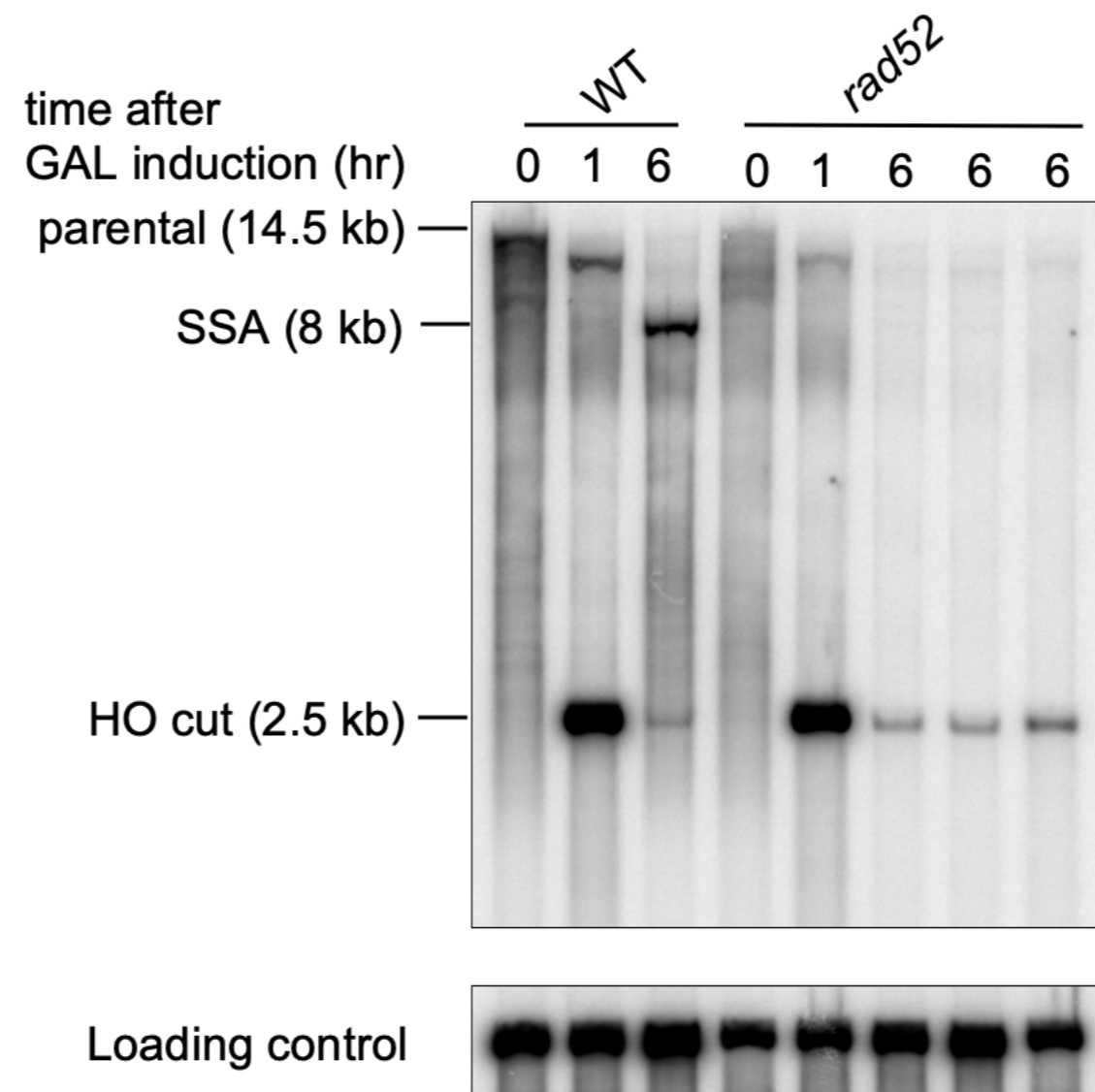

Supplement: S2 Fig — Representative southern blots showing DSB repair products by SSA in WT and rad52Δ strains. (PDF) [file pgen.1008689.s002.pdf]

S3 Fig

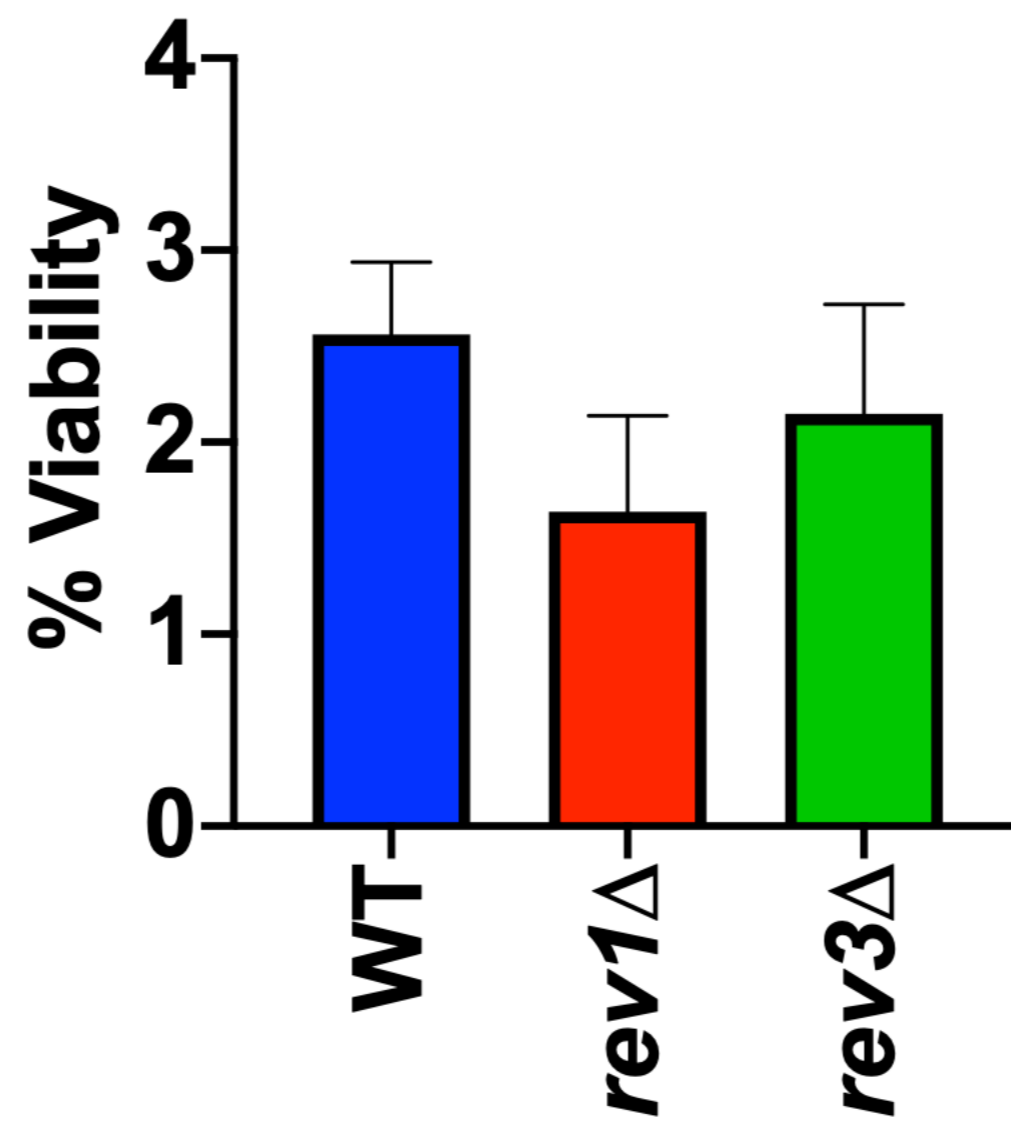

Supplement: S3 Fig — Cell viability following and HO-induced DSB with transformed ssODN with 37-nt of perfect homology and a 6-nt XhoI restriction site. n = 3. Error bars refer to standard error of the mean. (PDF) [file pgen.1008689.s003.pdf]

S4 Fig

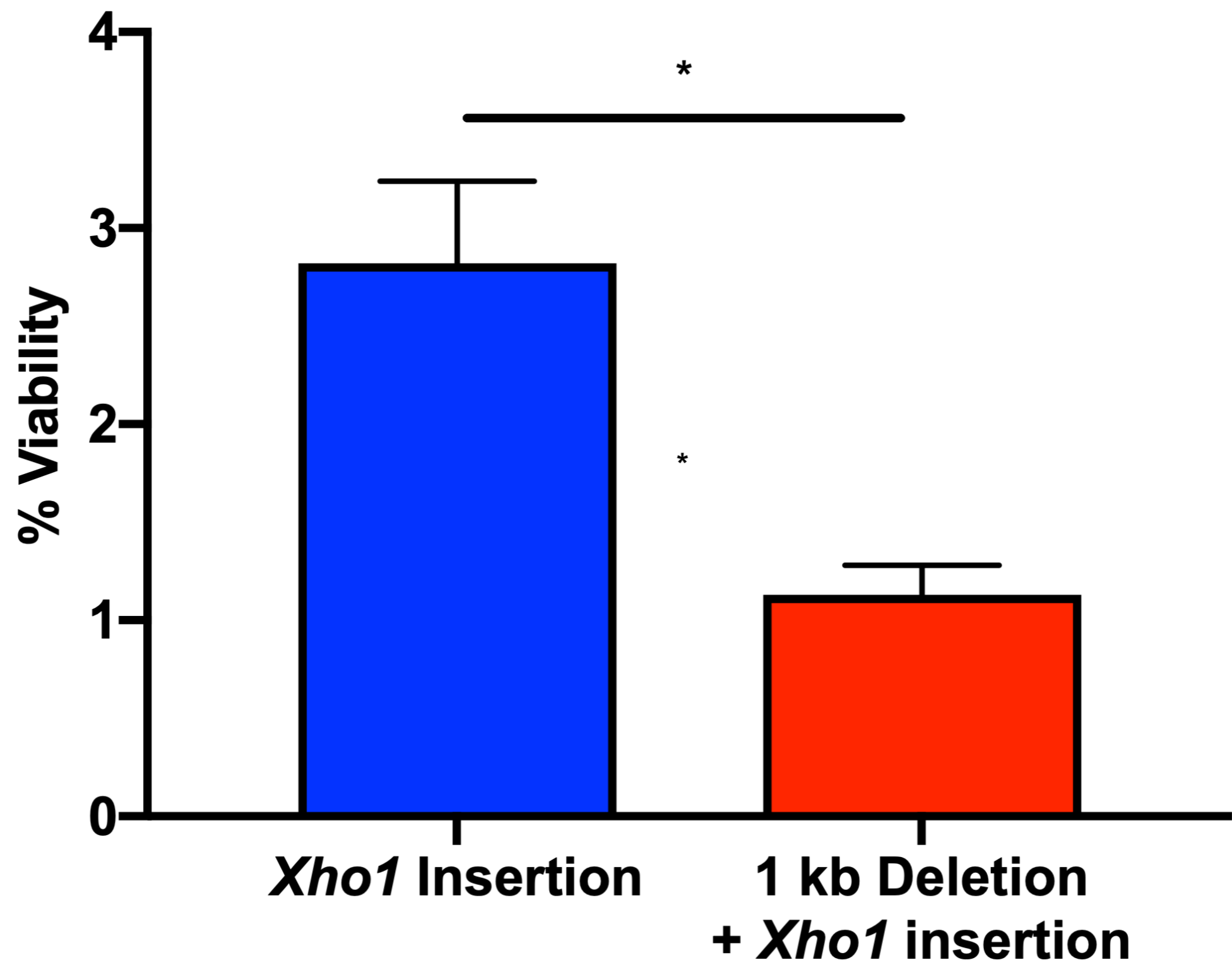

Supplement: S4 Fig — Cell viability following a DSB with transformed 80-nt ssODNs that create a 6-bp insertion versus a 1-kb deletion marked by a 6-bp insertion. Viability determined by colony counts of galactose-induction media over colony counts of YEPD non-induction media. Significance determined using a paired t-test, * p ≤ 0.01. XhoI insertion n = 19 (averaged across all assays), 1 kb deletion n = 8. Error bars refer to standard error of the mean. (PDF) [file pgen.1008689.s004.pdf]

**S5 Fig**

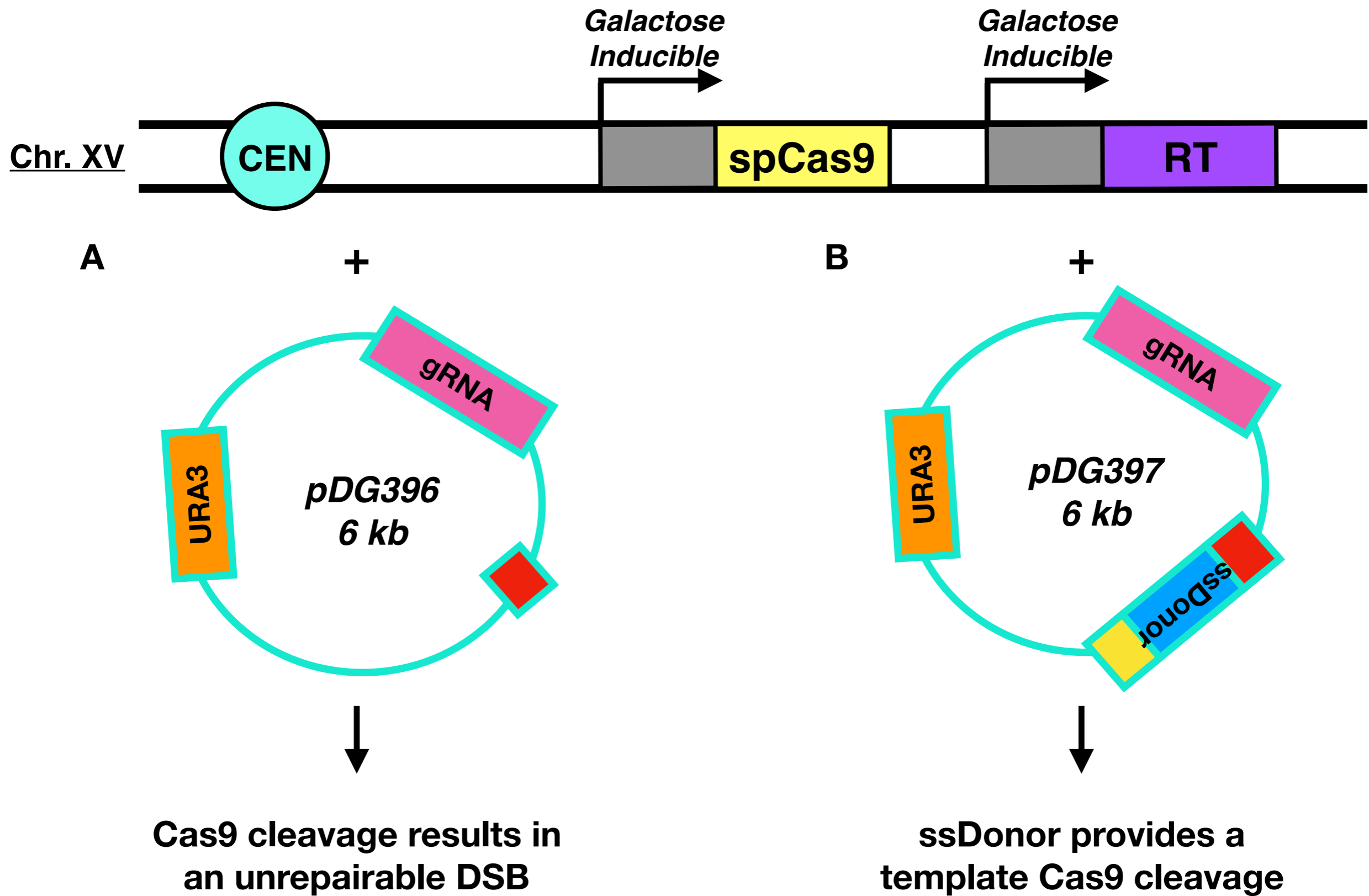

Supplement: S5 Fig — Galactose-inducible, yeast optimized spCas9 was introduced into the trp1 locus along with a galactose-inducible, yeast optimized retron, Ec86 (RT). Upon galactose-induction apo-Cas9 and the retron are transcribed. The blue region of the ssDonor (single-stranded donor) is the donor sequence to repair the DSB break, while the red region refers to a 34-bp consensus region that the retron binds to on the mRNA transcript to initiate reverse transcription, and the yellow region represents the termination sequence. The ssDonor and the gRNA are constitutively active. Galactose-induction results in A) and irreparable DSB since no donor is encoded, or B) repair of Cas9 cleavage via the reverse transcribed retron system. (PDF) [file pgen.1008689.s005.pdf]

S6 Fig

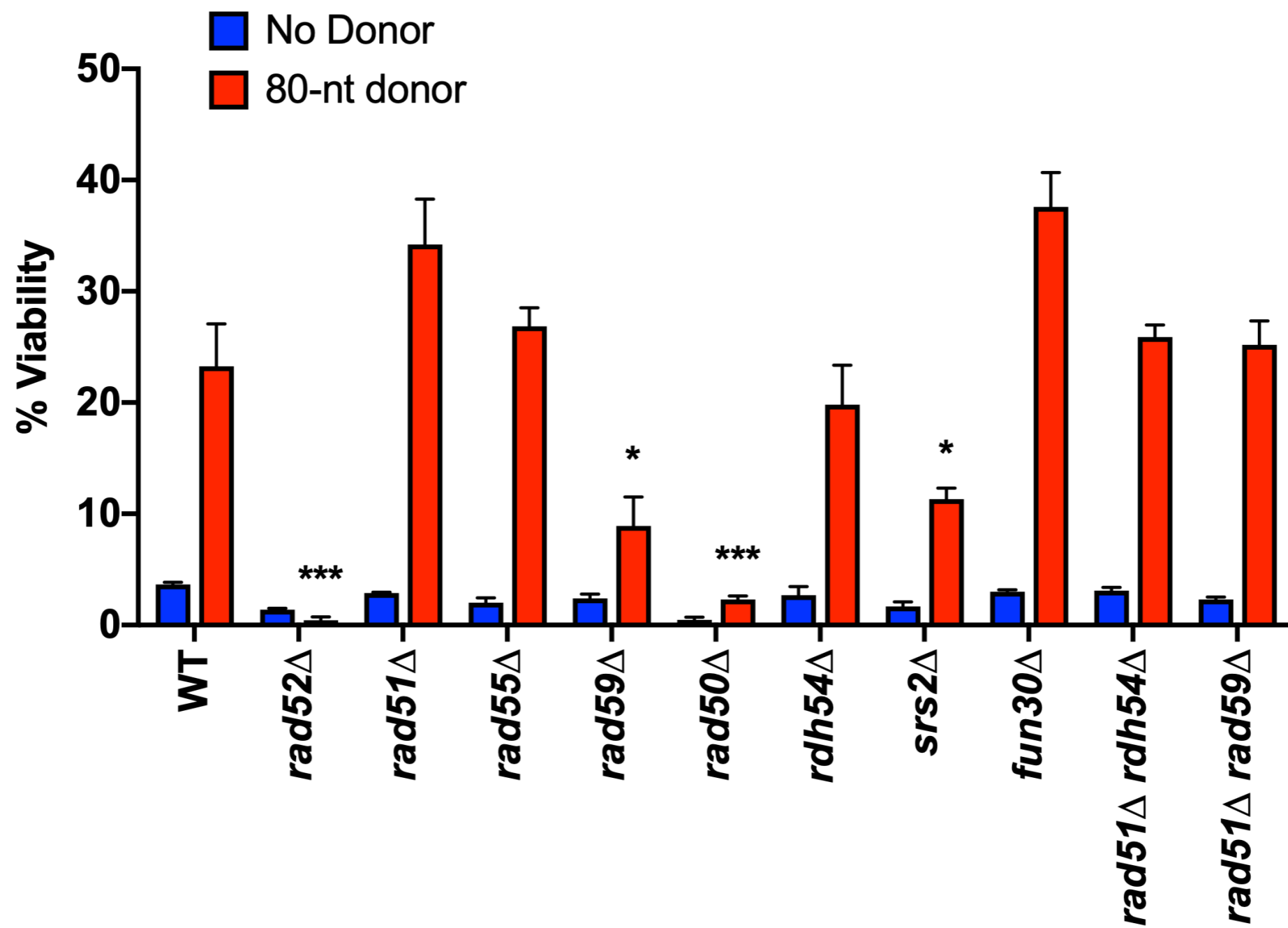

Supplement: S6 Fig — Upon galactose-induction, a Cas9-mediated DSB is created at MATα, which can be repaired through a retron-genereated ssDNA template, resulting in insertion of the XhoI restriction site. Colonies for each mutant were plated onto URA drop-out media with dextrose (non-induction) and URA drop-out media with galactose induction media. Plates were counted and the % Viability determined by average count of induction survivors over average count on non-induction media. Significance was determined using two-tailed t-tests compared to WT, using the two-stage Benjamini, Krieger, and Yekutieli false discovery rate approach [89], * p ≤ 0.01, ** p ≤ 0.001, *** p ≤ 0.0001. Error bars refer to the standard error of the mean. n = 3. (PDF) [file pgen.1008689.s006.pdf]
