# Supplementary material for: A Rad51-independent pathway promotes single-strand template repair in gene editing
Source: PLoS Genet. 2020 Oct 15;16(10):e1008689. doi: 10.1371/journal.pgen.1008689 (PMC7591047; doi:10.1371/journal.pgen.1008689)
Supplement: S1 Table — (DOCX) [file pgen.1008689.s007.docx]

| **S1 Table.** Strains used in these experiments | | |
| --- | --- | --- |
| **Strain** | **Genotype** | **Notes** |
| DG_24 | JKM179; *rad59::URA3* |  |
| DG_25 | JKM179; *rad51::URA3* |  |
| DG_26 | JKM179; *rdh54::KAN* |  |
| DG_27 | JKM179; *sgs1::URA3* |  |
| DG_29 | JKM179; *srs2::KAN* |  |
| DG_30 | JKM179; pJH627 |  |
| DG_31 | JKM179; *rad50::KAN* |  |
| DG_32 | JKM179; *mre11::KAN* |  |
| DG_33 | JKM179; *rad52::KAN* |  |
| DG_37 | JKM179; *rad54::NAT* |  |
| DG_38 | JKM179; *exo1::NAT* |  |
| DG_39 | JKM179; *sae2::NAT* |  |
| DG_40 | JKM179; *fun30::KAN* |  |
| DG_41 | JKM179; *mph1::NAT* |  |
| DG_42 | JKM179; *rad55::NAT* |  |
| DG_43 | JKM179; *pms2::KAN* |  |
| DG_44 | JKM179; *rad1::NAT* |  |
| DG_45 | JKM179; *pol2-4* |  |
| DG_46 | JKM179; *pol3-01* |  |
| DG_47 | JKM179: *rad51::URA3; rad59::KAN* |  |
| DG_48 | JKM179: *rdh54::KAN; rad59::URA3* |  |
| DG_49 | JKM179: *rdh54::KAN; rad51::URA3* |  |
| DG_50 | JKM179: *rad51::URA3; srs2::NAT* |  |
| DG_51 | JKM179: *rdh54::KAN; srs2::NAT* |  |
| DG_52 | JKM179: *sgs1::URA3; exo1::NAT* |  |
| DG_78 | *ho*Δ *MAT*α *hml*Δ::*ADE1* *hmr*Δ::*ADE1 ade1-100 leu2-3,112 lys5* *trp1::Gal::spCas9::Gal::Ec86::trp1* *ura3-52* |  |
| DG_79 | DG_78; *rad51::HPH*; pDG396 |  |
| DG_80 | DG_78; *rad52::KAN*; pDG396 |  |
| DG_81 | DG_78; *rad55::NAT*; pDG396 |  |
| DG_82 | DG_78; *rad59::NAT*; pDG396 |  |
| DG_84 | DG_78; *rad50::NAT*; pDG396 |  |
| DG_85 | DG_78; *rdh54::NAT*; pDG396 |  |
| DG_86 | DG_78; *srs2::NAT*; pDG396 |  |
| DG_87 | DG_78; *fun30::NAT*; pDG396 |  |
| DG_88 | DG_78; *rad51::HPH*; pDG397 |  |
| DG_89 | DG_78; *rad52::KAN*; pDG397 |  |
| DG_90 | DG_78; *rad55::NAT*; pDG397 |  |
| DG_91 | DG_78; *rad59::NAT*; pDG397 |  |
| DG_92 | DG_78; *rad50::NAT*; pDG397 |  |
| DG_93 | DG_78; *rdh54::NAT*; pDG397 |  |
| DG_94 | DG_78; *srs2::NAT*; pDG397 |  |
| DG_95 | DG_78; *fun30::NAT*; pDG397 |  |
| DG_104 | JKM179: *URA3-MX* | *URA3*-MX integrated 200 bp upstream HO cut site |
| DG_105 | DG_104; *rev1::NAT* |  |
| DG_106 | DG_104; *rev3::NAT* |  |
| DG_109 | DG_78; *rdh54::KAN; rad51::HPH*; pDG396 |  |
| DG_110 | DG_78; *rdh54::KAN; rad51::HPH*; pDG397 |  |
| DG_112 | DG_78; *rad59::NAT rad51::HPH*; pDG396 |  |
| DG_113 | DG_78; *rad59::NAT rad51::HPH*; pDG397 |  |
| DG_115 | JKM179; *mre11::KAN; ku70::NAT* |  |
| DG_116 | JKM179: *URA3-MX* | *URA3*-MX integrated 200 bp downstream HO cut site |
| DG_117 | JKM179: *URA3-MX* | *URA3*-MX integrated 500 bp upstream HO cut site |
| DG_118 | JKM179: *URA3-MX* | *URA3*-MX integrated 1000 bp upstream HO cut site |
| DG_119 | JKM179: *URA3-MX* | *URA3*-MX integrated 1500 bp upstream HO cut site |
| DG_120 | JKM179: *URA3-MX* | *URA3*-MX integrated 2000 bp upstream HO cut site |
| yMV45 | *ho hml::ADE1 MATa::hisG hmr::ADE1 leu2::leu2(Asp718-SalI)-URA3-*pBR332*-MATa ade3::GAL::HO ade1 lys5 ura3-52 trp1::hisG* | (Vaze et. al., 2002) |
| NP_706 | yMV45; *rad51::KANMX* |  |
| NP_558 | yMV45; *rad59::NATMX* |  |
| NP_707 | yMV45; *rad51::KANMX rad59::NATMX* |  |
| NP_591 | yMV45; *rad52-R70A* |  |
| NP_708 | yMV45; *rad51::KANMX* *rad52-R70A* |  |
| NP_610 | yMV45; *rad59::NATMX rad52-R70A* |  |
| NP_709 | yMV45; *rad51::KANMX*  rad59*::NATMX rad52-R70A* |  |
| NP_867 | yMV45; rad52*::HPHMX* |  |
