# Supplementary material for: A Rad51-independent pathway promotes single-strand template repair in gene editing
Source: PLoS Genet. 2020 Oct 15;16(10):e1008689. doi: 10.1371/journal.pgen.1008689 (PMC7591047; doi:10.1371/journal.pgen.1008689)
Supplement: S2 Table — (DOCX) [file pgen.1008689.s008.docx]

| **S2 Table.** Oligonucleotides used in these experiments | | |
| --- | --- | --- |
| **Oligo** | **Sequence** | **Description** |
| DG_90 | CAGCTCCATTGCGTATCATGTCCTTTGCTATCGCGTGTGCTGGTAGGATTGGCGTCTTTCCGGAACCTGAATACGATCCC | Repair oligo to introduce *pol3-01* mutation via Cas9 |
| DG_91 | TTCATTGAAGATACTAGGAAAATTGCATTTGCCGATCCAGTAGTGATGGCTTTCGCAATAGCTACCACGAAGCCGCCTTTAAAATTCCCGGATTCCGC | Repair oligo to introduce *pol2-4* mutation via Cas9 |
| DG_253 | ACGTCTAGCTGAGCATGTGA | Sense oligo in Y region of *MAT*⍺. Anneals 327 bp upstream of HO cut site |
| DG_254 | TACGTTAAACATGAATCGA | Reverse oligo in Taf2. Anneals 1869 bp downstream of HO cut site |
| DG_285 | GATGTCTGGGTTTTGTTTGGGATGCAATTTATT  GCTTctcgagTCGGCTTCACAATTTGTTTTTCC  ACTTTTCTAACAGG | 1 kb deletion donor - inserts *Xho*I site at *MAT*⍺ after HO-induced DSB and resection |
| DG_287 | AAAATGCAGCACGGAATATGGGACTACTTCGCGCAACctcgagAGTATAATTTTATAAACCCTGGTTTTGGTTTTGTAGA | 100% homology donor – inserts *Xho*I site at *MAT* after HO-induced DSB |
| DG_310 | TCTACAAAACCAAAACCAGGGTTTATAAAATTATACTctcgagGTTGCGCGAAGTAGTCCCATATTCCGTGCTGCATTTT | Reverse complement of DG_287 to create dsDNA template |
| DG_344 | ATCTGAGTTACTGTCTGTTTTCCTTCTGCTCGCTGAAGAATGGCACGCGGACAAAATGCACTCGAGGCACGGAATATGGGACTACTTCGCGCAACAGTATAATAGGAAACCCGTTTCTTCTGACGTAAGGGTGCGCACACGCGGACAAAATGCAGCAGTTTCAGAGCTATGCTGGAAACA | *MAT*⍺.80-nt retron donor sequence to repair *MAT* and insert *Xho*I insertion |
| DG_373 | AcAATGCAGCgCGGAATATaGGACTACTgCGCGCAACctcgagAGTATAATTTTATAAACCCTGGTTTTGGTTTTGTAGA | Inserts *Xho*I site at *MAT*⍺ after HO-induced DSB. Mismatches in homology every 9^th^ bp on the 5’ side |
| DG_374 | AAAATGCAGCACGGAATATGGGACTACTTCGCGCAACctcgagAGTATAATgTTATAAACtCTGGTTTTaGTTTTGTAtA | Inserts *Xho*I site at *MAT* after HO-induced DSB. Mismatches in homology every 9^th^ bp on the 5’ side |
| DG_396 | CGGCATCCTGCATTGAATCTGAGTTACTGTCTGTTTTCCTAGGAAACCCGTTTCTTCTGACGTAAGGGTGCGCAATGAGTTTACGTTCGAGGCGGTTTCAGAGCTATGCTGGAAACAGCATAGCAAGTTGAAAT | gBlock to clone into pZS165. gRNA to target 5bp insertion of *lys5* in JKM179  No retron donor sequence |
| DG_397 | CGGCATCCTGCATTGAATCTGAGTTACTGTCTGTTTTCCTTTCAAGAGGATATACTCGCGGATGAGTTTACGTTCGAGGCATTAATGAGAACTTTGCCATTGGCGTCTCAAGCCAGAATCAGGAAACCCGTTTCTTCTGACGTAAGGGTGCGCAATGAGTTTACGTTCGAGGCGGTTTCAGAGCTATGCTGGAAACAGCATAGCAAGTTGAAAT | target 5bp insertion of *lys5* in JKM179  80-nt retron donor sequence to restore Lys5 to prototrophy |
