# Supplementary material for: A Rad51-independent pathway promotes single-strand template repair in gene editing
Source: PLoS Genet. 2020 Oct 15;16(10):e1008689. doi: 10.1371/journal.pgen.1008689 (PMC7591047; doi:10.1371/journal.pgen.1008689)
Supplement: S3 Table — (DOCX) [file pgen.1008689.s009.docx]

| **S3 Table.** Plasmids used in these experiments | |
| --- | --- |
| **Plasmid** | **Description** |
| pDG_344 | pZS165 with gRNA to target *MAT*⍺ (CACGCGGACAAAATGCAGCA) with 80 nt retron donor sequence to repair Cas9 DSB (TCTGCTCGCTGAAGAATGGCACGCGGACAAAATGCActcgagGCACGGAATATGGG  ACTACTTCGCGCAACAGTATAATA) |
| pDG_396 | pZS165 with gRNA to target *lys5* (ATGAGTTTACGTTCGAGGCG) with no retron donor sequence |
| pDG_397 | pZS165 with gRNA to target *lys5* (ATGAGTTTACGTTCGAGGCG) with 80 nt retron donor sequence to repair Cas9 DSB (TTCAAGAGGATATACTCGCGGATGAGTTTACGTTCGAGGCATTAATGAGAACTTTG  CCATTGGCGTCTCAAGCCAGAATC) |
| pRA_114 | Cas9 vector used to make *pol3-01* (guide TCCTTTGATATCGAGTGT GC) |
| pRA_124 | Cas9 vector used to make *pol2-4* allele (guide TATCAAATGCCATTAC CACA) |
| PL_634 | Cas9 vector used to make *rad52-R70A* allele (guide ACTCTCTTGGAGATATACTC) |
