# Supplementary material for: A Rad51-independent pathway promotes single-strand template repair in gene editing
Source: PLoS Genet. 2020 Oct 15;16(10):e1008689. doi: 10.1371/journal.pgen.1008689 (PMC7591047; doi:10.1371/journal.pgen.1008689)
Supplement: Attachment — Submitted filename: SSTR Response to Reviewer Comments.docx [file pgen.1008689.s011.docx]

Reviewer's Responses to Questions

**Comments to the Authors:
Please note here if the review is uploaded as an attachment.**

Reviewer #1:
*1. The SSA data do not fit well with the other analyses, so perhaps should be moved to the supplement or omitted altogether. The main thing shown is that suppression of rad59D by rad51D requires the Rad52 annealing activity. Why not just show this with the SSTR system?*

We strongly disagree that these data should be moved to the supplement. That Rad59 exerts a Rad52-dependent function on strand-annealing and SSTR, that is suppressed by deleting Rad51 is an important new finding. We feel that the SSA data pinpoint when we are seeing the effect of Rad59 in the SSTR system. Thus, the SSA data are important to retain in the paper. We have reorganized the data to group all of the *rad51*Δ *rad59*Δ data in Figure 2.

*2. Panels E and F in Fig. 1 are out of place – not described until after the SSA data in Fig. 2.*

These data (and additional data on target-adjacent mutagenesis) have been moved to create a new figure 3

*3. In Figure 5C, the colors of WT and the mismatch-containing ODN are reversed relative to panel A, which makes things unnecessarily confusing.*

The color scheme of 5C has been changed to match 5A

*4. In Figure 5B, any idea why viability increases in the absence of proofreading activity?*

Based on our previous studies (Tsaponina et al., 2013), we can surmise that the polymerase may be more processive and complete the replication steps more often. However, the differences are not statistically significant.

*5. The text in Figure 6 needs some editing: the first point under (A) is confusing and there is no “invading” strand as stated in panel (B).*

We agree that this nomenclature is confusing. We have replaced “invading” with “annealed recipient.”

*6. How many repair events were sequenced for Figure 7A? If only 23 were sequenced from strain and each mismatched ODN, then the % are likely not very accurate. The error for each proportion can be determined using Vassarstats.*

The differences we show are very different qualitatively – from the majority of events on the 3’ side having conversions to almost none; for sites 2 and 3, where Pol3 apparently doesn’t contribute, 14/23 events have one or both sites conversions, whereas none have full conversions with *msh2*∆. Similarly on the 5’ side, sites 2 and 3 go from 12 one or both sites conversions and 3 heteroduplexes to 2 conversions and 17 heteroduplexes. By contingency chi-square tests these are significantly different.

*7. Might the asymmetry in the DSTR system reflect a transcription-generated asymmetry of the broken ends?*

As this data is inconclusive and doesn’t contribute to overall study of SSTR, Figures 8D and 8E have been removed from the manuscript

*8. Line 459 states that the gap-filling (when creating a large deletion) depends on Pol zeta. Where was the demonstrated? There was not a rev3 mutant in Figure 4. The only thing shown to be dependent on Pol zeta was URA3-associated mutagenesis, which likely involves limited synthesis to bypass DNA damage that accumulates in ssDNA.*

We apologize that the paragraph was misleading. The statement about gap-filling was not intended to focus on the situation where a large deletion was generated, and where we have not examined target-adjacent mutagenesis, but rather more generally. The paragraph has been re-worded accordingly.

*9. It is very striking that Sae2 has no effect in MRX-mediated resection. Any explanation?*

The MRX complex has many functions beyond resection, including end-bridging, the recruitment of cohesion to sites of DSB damage, facilitating sister-chromatid recombination as well as being required for nonhomologous end-joining. Deleting *SAE2* doesn’t affect end-joining and has a less-profound effect on resection of HO ends than deleting MRE11 or RAD50 (for example, in G2/M-arrested cells deleting MRE11 or RAD50 blocks resection whereas deleting SAE2 doesn’t). This is a long way of saying that we don’t know what step(s) involve MRX, but whatever step it is, doesn’t need Sae2. We now make this point.

*10. In most Figures, it needs to be stated what the error bars correspond to. Also, it needs to be stated how many times were experiments repeated. How does a paired t-test differ from a normal t-test and why was it used?*

The error bars refer to the standard error of the mean and has been stated so in the figure legends, although not explicitly in the text. These data were analyzed using an unpaired two-tailed t-test. Most of these data (Figures 1, 2a, 4 and 6) also use Welch’s correction. WT and *rad51*𝛥 were used as controls in every experiment, and therefore have a much larger sample size than most mutants tested which were repeated 3 times each.
 *11. The effect of mismatches on editing efficiency is relegated to the supplement. Why not incorporate into Figure 5 (see Figure 7B)?*

Fig S4 has been incorporated into Figure 6

*12. Is it really necessary to continue showing the NHEJ in all Figures? Only SSTR or DSTR is discussed in the text.*

We thank the reviewer for ensuring that we discuss the NHEJ outcomes, which in some cases account for nearly all the survivors. Showing both types of repair are important for clarity

Line 81 – delete “the fate of”
Line 117 – all survivors “in the rad52D background” resulting
Line 224 – duplex DNA?

The text has been adjusted to reflect these comments.

Reviewer #2 Comments:

*A previous study by Storici et al (2006) found a lower efficiency of SSTR in the rad51 rad59 double mutant than observed for the single mutants. Do the authors have an explanation for the discrepancy between the studies?*

We do not have an explanation for the discrepancy between the studies. However, in the Storici paper they used I-SceI to create the DSB which could account for the difference. Our data are consistent with the SSA data and we are confident they are correct both for HO and Cas9-induced DSBs.

*Is the requirement for MRX suppressed by eliminating Ku? This might help address whether the MRX defect is due to the resection function or another activity of the complex.*

We have now shown that deleting Ku doesn’t suppress the severe defect in *mre11*∆

*Figs 1, 3, 5, 6, 7 and 8: How many independent trials were performed for each strains? Also, the error bars need to be defined.*

This has been addressed above.

*Fig 1E and F: these data do not really fit here and should be in a supplementary figure. Several other studies have shown mutagenesis of a nearby URA3 marker during DSB repair as a result of resection and fill-in synthesis by Pol zeta. I understand that the authors are making the point that investigators using SSTR to correct mutations in mammalian cells need to check the flanking sequence. If these are kept in it would be useful to test the distance dependence for mutagenesis.*

This experiment has been expanded to look at mutagenesis at different distances. We find the mutagenic effect is localized within about 1 kb of the site of the DSB. In addition we have shown that similar events occur with dsDNA as well as ssDNA templates, though we do not know if these differences are important. These data have been made into a new figure 3. Work from the Strathern lab and others has been cited in the text.

*Figs 1 and 4: Figs 1 and 4 could be combined, just showing the differences in genetic requirements when SSTR is used to create a deletion (i.e., the rad1 and exo1 sgs1 data).*

The dsDNA data are now included in Figure 1.

*Fig 2A: What is the length of the direct repeats? The SSA data appear to have been added to support the conclusion about the Rad59 role, but don't fit with the rest of the study. If Fig 2 is to be included, the authors would need to test rad52-R70A and rad51 rad52-R70A in the SSTR assay, and show the rad52 null in the SSA assay.*

This has been clarified in the text (revised Fig. 2) and the experiments have been repeated in the SSTR assay to confirm that *rad52-R70A* is SSTR-defective and not suppressed y deleting Rad51..

*Fig 3C: Are the p values for comparisons of mutants to WT? What is the p value for rad59 vs rad51 rad59? Correct 80 bp donor to 80-nt.*

As stated in the Figure legend (now Fig. 5), all p-values compare the mutant to the wt, which has been stated in the figure legends. However, there is also a significant difference between rad59𝛥 and rad59𝛥rad51𝛥. This has been added to the Figure legend. 80 bp has been corrected to 80-nt.

*Fig S4: It would be better to have these data included in Fig 5. The pol2-4, pol3-01, sgs1 and msh2 ssODN targeting efficiency should also be done with the homologous template.*

Fig S4 has been incorporated into Figure 6. Sgs1 has been shown to not be significantly different from WT with the homologous template in Figure 1. Also, *sgs1*Δ, *pol2-4* and *pol3-01* have been added to 6b

*Fig 6. The text at the bottom does not make sense as written. The left side of figure shows assimilation of the mismatches not removal. For the right panel, the statement that in the absence of Msh2 there should be no inheritance is incorrect because Fig 5 shows that mismatches can be incorporated in the msh2 mutant.*

We have re-worded our conclusion to agree with the reviewer that there still is inheritance of the mutations in the absence of Msh2, but they come from heteroduplex DNA, resulting in mixed colonies. Figure 5 shows sectored colonies in the absence of msh2 – they are left as a heteroduplex which means they are neither fully incorporated nor corrected to the original sequence. This indicates that Msh2 is responsible for fully incorporating the edits into the genome.

*Fig 7A,B would work better incorporated into Fig. 5 with the model (Fig 6) coming afterwards.*

These data have been moved

*Fig 8B, C: these are labeled SSTR instead of DSTR. There is only ~50% decrease in DSTR in the rad51 mutant; could this be due to resection of the template to make it ssDNA and therefore switching to Rad59 dependence/Rad51 independence? I think it would be more appropriate to have the SSTR/DSTR comparison included in Fig 1 or as Fig 2.*

It is possible that the dsDNA template is being resected or unwound into ssDNA. It is also possible (and we think more likely) that there is a background of ssDNA template after S1 treatment. If the dsDNA template is not treated with an S1 nuclease we see that there is not a requirement for Rad51, indicating that ssDNA is prominent in the annealed template pool. This has been stated in text.

These data have been moved to be part of figure 1.

*Fig 8E, F: These data could be removed, they are inconclusive and do not add to the conclusions of the study.*

Figure 8D and E have been removed.

*Line 381: Could the authors explain what they mean by “distinct from that involving gene conversion between long regions of homology”?*

We are referring to traditional DSB repair and it’s genetic requirements.

Reviewer #3 Comments:
*1. In Fig 1, the statistical (in)significance is confusing. For sgs1 and rad59, mutation of either seems to reduce SSTR substantially, yet “in subsequent assays Rad59 proved to be required”. On the other hand, Sgs1 is concluded not to be important, although rad59 and sgs1 mutants both seem to have -fold reductions in SSTR. Also, mre11 does not appear to be significant, despite the large effect and reasonable small error bars (SD or SEM). How many experiments were performed?*

Each experiment was performed 3 times. Statistical analysis has been redone using t-tests and we have concluded that the reduction in viability of *rad59*Δ meets our p-value cut off of 0.01, however *sgs1Δ* (p = 0.014) still does not meet our cut off requirements. We feel it is important to use such a stringent cut-off for significance since so many mutants are being compared. We have adjusted the text to clarify this.

*2. Although the SSA assay is useful (Fig 2), it is difficult to extrapolate to SSTR. For example, SSTR is increased 4-fold in the rad51rad59 double mutant, but SSA is only rescued somewhat. Is there a reason why SSTR was not examined in the rad52-R270A mutant, to try to address the genetic relationship? The authors should consider moving this figure to the supplement, since it is something of a distraction.*

This has been addressed in previous comments.

*3. The retron results are impressive. However, the authors conclude that the SSTR genetic requirements are identical using Cas9 and HO (line 189). While broadly similar (e.g., Rad52 dependence; Rad51 independence), there are some clear distinctions (Fig 3C). For example the dependence for on Srs2 is greatly reduced and the effects of rad51 on rad59 and rdh54 are greatly muted. There are other key differences in the analysis with Cas9 besides the nuclease, eg, use of the retron system. Therefore, the conclusion needs to be tempered. The authors should directly compare Cas9 and HO using the same ssODN approach at the same locus to get some understanding of what the differences are. (Is it not possible to target Cas9 – or a Cas9 with a modified PAM - to the HO site?)*

Although it is possible to engineer a PAM into the *MAT* locus, the AT-rich nature of this region cause poor Cas9 cleavage efficiency. We have used multiple approaches to try these experiments with Cas9 and an ssODN, but there is an issue of efficiency in getting enough ssODN past the cell wall to get enough survivors to do a quantitative analysis. For these reasons we had to move to the retron system.

We have now repeated the retron experiments at the *MAT* locus, which have been added to the supplement. These results fully support our conclusions.

*4. Fig 1E. What happens to mutagenesis frequency if the URA3 gene is placed 3’ to the ssODN site? Or if a dsDNA template is used?*

These experiments have been performed and are shown in Figure 3

*5. For greater clarity, a suggestion would be to reorganize Figures 5 and 6 such that each figure contains one substrate (5’MM or 3’MM), the observed products, and the models for wildtype and for msh2. For example, with 5’MM, it would seem like MSH2 works on both strands, given the predominance of heteroduplex products in the mutant and the presence of a substantial number of products with both 3 (10 products) and no incorporated polymorphisms (7 products), but that is not apparent in the model.*

These figures have been reorganized, but we think it is important to show the 5’ and 3’ MM templates side by side so that it is clear that edits on either side of the DSB have different effects. Although we do see some edits and heteroduplexes in *msh2*Δ, it is a significant reduction from the WT. Therefore, we believe that Msh2 acts at the annealing step on the 3’ side, but at the correction step on the 5’ side of the DSB.

*6. The authors need to discuss the new results from yeast with previously published results. Although the references are there, a coherent discussion is lacking.*

The text has been adjusted and we trust it is more clear.

Additional comments:

*Abstract: The authors should consider a punchier abstract to more succinctly get across key points.*

We hope we have done so. *Line 104: It would be helpful to indicate here, rather than below, the 10-fold improved survival by adding the ssODN: 2.8% (line 108) vs 0.2% (line 97), although NHEJ rang from 10-25% (line 104-105)

Fig 1B. Error bars should be added for the NHEJ events in each bar.*

The error bars refer to the viabilities. The NHEJ vs SSTR was determined by pooling survivors and subsequent PCR and restriction digest and quantification via a gel doc, discussed in the figure legends and methods and Figure S1.

*Line 121: Specify which Rad51-independent pathways.*

We have adjusted the text. *Line 124: The distinction for Sae2 is interesting. What is the Sae2 requirement for other Rad51-independent pathways?*

The role of Sae2 has not been tested in most Rad51-independent pathways, but we have added to the text about potential roles of Sae2. *Line 130: It’s clear that rad51rdh54 are not epistatic (Fig. 1D); however, the epistasis analysis was not done together with srs2 (Fig. 1C) the focus of this section.*

The figures and text have been adjusted. *Line 131 rad51rdh54 (Fig 1D): 4-fold not 7-fold? If 7-fold, please clarify.*

This has been corrected. *Line 142: It would appear that deletion of Rad59 has a much smaller effect than rad52 R270A (Fig. 2) not the “similar” effect that is stated. Is the difference not significant, despite appearances?*

Significance has been added to the figure.

*Line 154: “Deleting Sgs1 had no effect” same comment as above. Also, this is Fig. 1C not 1D*

We have specified that Sgs1 has no statistically significant difference.

*Fig 5: the color scheme is confusing. In A the ssODN is black and the chromosome red, but in C the ssODN sequences incorporated are red.*

*This has been corrected

Line 308: Only the mutation 2-nt from the 3’ end of the donor is said to be incorporated at a higher frequency in the pol3-01 mutant. However, it appears that that mutations in the 5’MM are incorporated at a somewhat higher rate. Is this not significant?*

Since only 23 SSTR events were sequenced for each template per strain, we do not feel like we can make any strong quantitative conclusions on these data, with the exception of Pol3 since it has such a strong qualitative phenotype that shows significance via Chi Square analysis.

*Line 312: it’s not clear why the authors suggest that the ssODN could be directly incorporated in some cases. Why not just propose a model as in 6B where the last mutation is maintained in a pol3-01 background now simply be copied like the rest of the 3’ mutations?*

We do not believe that the ssODN is synthesized on the 3’ end because it is the only side of the ssODN that is able to anneal to the resected DNA.

*Line 326: Please explain what inverted thymine/dideoxythymines are.*

We have adjusted the text to clarify what the inverted thymines/dideoxythymines are used to block annealing and elongation of the the ssODN *Line 346: Is another acronym necessary since DSTR seems to have the genetic dependencies of classical HR?*

SSTR is a form of HR, and we feel that labeling the DSTR events as HR would give the wrong impression.

*Fig 8C,D. are the panels mislabeled – SSTR?*

These figures have been removed from the manuscript

*Line 357: it’s confusing to switch to “9 bp upstream” or “9 bp downstream” when the mutations are specifically numbered.*

The mismatches are both numbered as “1” and are two different templates. We believe that referring strictly to the numbers would make the text more confusing, and also take away from the emphasis of the section, which is that mismatches templated upstream or downstream of the DSB are not incorporated into the genome in the same manner.

*Line 363: Given the ambiguity, consideration should be given to moving these figure panels (8D,E) to the supplement.*

These figures have been removed from the manuscript

*Line 426: The language is not always precise and this is one example. “The initial annealing of the resected end with the template…” There are two resected ends and moreover the authors shift between ssODN, template, and donor”. Why not change around the sentence to “The initial annealing of the ssODN with the complementary resected end…”

Fig. 6: What prevents extension of the donor? Or is this considered irrelevant since it would be dissociated?*

We believe that the donor can be extended by Pol3, which is stated in the text

*Line 438: inconsistent “…all of the heterologies will be copied” but line 441 “The failure to incorporate..” Delete “all of”*

The text has been adjusted *Line 454: Fig 8 not Fig 7?*

The data has been reorganized and the text adjusted

**Have all data underlying the figures and results presented in the manuscript been provided?**
Large-scale datasets should be made available via a public repository as described in the *PLOS Genetics* [data availability policy](http://journals.plos.org/plosgenetics/s/data-availability), and numerical data that underlies graphs or summary statistics should be provided in spreadsheet form as supporting information.

Reviewer #1: Yes

Reviewer #2: Yes

Reviewer #3: No: A data file should be made available for the primary data for each of the figure panels (Unless I overlooked it)
